# Minimal invasive treatment of liver and kidney metastasis in mucosal melanoma of the right inferior turbinate

**DOI:** 10.1016/j.radcr.2024.09.014

**Published:** 2024-09-25

**Authors:** Pietro G. Lacaita, Reto Bale, Paolo Lucciarini, Van Anh Nguyen, Martin Freund, Elke R. Gizewski, Daniel Putzer

**Affiliations:** a Department of Radiology, Landeskrankenhaus Innsbruck- Medical University Innsbruck, Innsbruck, Austria; b Department of Visceral, Transplant and Thoracic Surgery (VTT), Landeskrankenhaus Innsbruck- Medical University Innsbruck, Innsbruck, Austria; c Department of Dermatology, Venereology and Allergology, Landeskrankenhaus Innsbruck- Medical University Innsbruck, Innsbruck, Austria

**Keywords:** Nasal cavity mucosal melanoma, Computed tomography (CT), Stereotactic radiofrequency ablation (SRFA), Interventional radiology, Endovascular embolization

## Abstract

This case report describes an 80-year-old female patient who initially presented with nasal epistaxis. The patient had a history of atrial fibrillation and arterial hypertension. Computed tomography of the facial sinuses revealed a large mass in the inferior right turbinate with slight expansion into the maxillary sinus. Endoscopic excision of the right nasal cavity was performed, and the histologic workup revealed mucosal melanoma of the nasal cavity (cT3, cN0, cM0). A medial maxillectomy of the right side, including 2 biopsies within 1 month, showed no signs of recurrence. After 1 year, the patient was diagnosed with liver and renal metastases in a follow-up CT, which were treated with stereotactic radiofrequency ablation. After spending 2 weeks in the intensive care unit due to postoperative complications, the patient recovered and was discharged from the hospital in good condition. A promising alternative minimally invasive therapeutic strategy, highlighted by our case, should be considered as a primary goal of tumor reduction.

## Case report

**Fig. 1:**
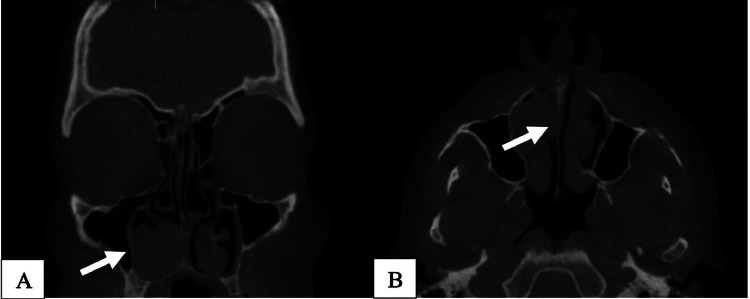
(A-B): CT findings of the facial sinus (Fig. 1A; left; coronal view) Expansive growing soft tissue mass of the inferior right turbinate measuring 2, 5 cm. No invasion of the skull or orbit is seen. (Fig. 1B; right; axial view) No signs of infiltration into other sinusoidal structures or bony erosion. Slight deviation of the nasal septum.

We present the case of an 80-year-old female patient with a history of atrial fibrillation and arterial hypertension under anticoagulant therapy. The patient initially presented to the ENT department complaining of recurrent unilateral epistaxis. No other symptoms were reported. Computed tomography (CT) of the facial sinuses showed an expansive, growing soft tissue mass of the inferior right turbinate measuring 2.5 cm with a slight deviation of the nasal septum and extension into the maxillary sinus (Figs. 1A and B).

CT showed no signs of infiltration into other sinusoidal structures or bone invasion.

An endoscopic excision and biopsy of the right nasal cavity were performed, which revealed changes suspecting a mucosal melanoma (MM) of the nasal cavity, classified as cT3 cN0 cM0.

Immunochemical analysis revealed strong positivity for protein S-100, Melan A, vimentin, and HMB-45. NRAS positive mutation and KIT, BRAF negative.

A medial maxillectomy of the right side was performed 1 month later, without evidence of malignancy upon histologic work-up. Several close follow-ups within a 3-month interval with subsequent endonasal endoscopy of the paranasal sinus and re-biopsy, as well as a whole-body CT after 6 months, showed no signs of recurrence. However, 12 months after the initial CT, new lesions were identified in the liver and kidney: a slightly hypervascularized hypodense lesion measuring 2.4 cm in segment VIII of the liver and another in the right upper pole of the kidney measuring 1.6 cm (Figs, 2A and B).

**Fig. 2:**
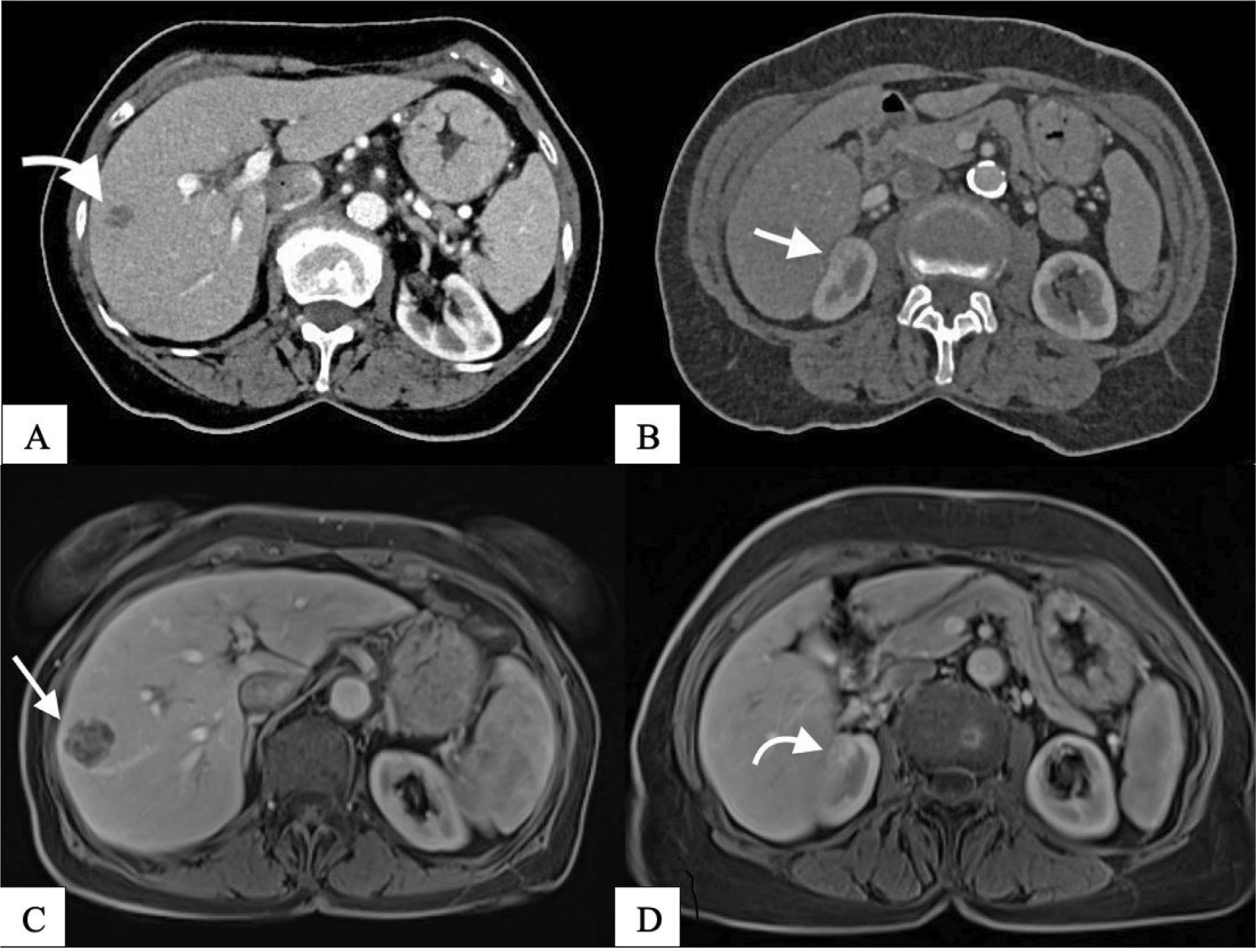
(A-D): Abdomen-CT and MRI 12 months after the initial diagnosis. 2A; upper left; axial view: slightly vascularized hypodense lesion on the segment VIII of the liver with a transversal 2, 4 cm. 2B; upper right; axial view: On the superior pole of the kidney a vascularized lesion with a 1, 6 cm. 2C; lower left; axial view: there is a pathological enhancement on the solid subcapsular mass. 2D; lower right; axial view: hypervascular lesion on the superior pole of the kidney with ca 1, 7 cm.

**Fig. 3:**
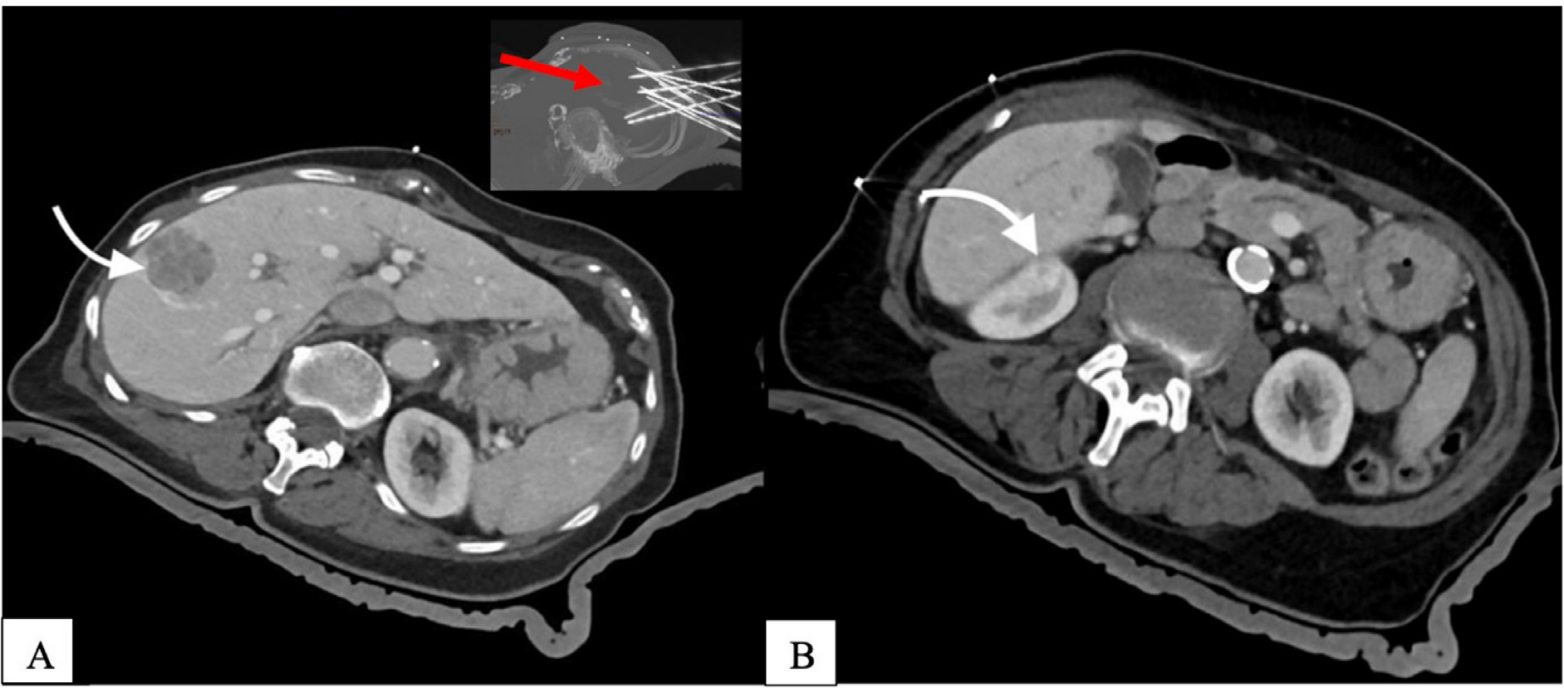
(A-B): Planning CT scan in arterial phase of the SRFA. (Fig. 3 A-B left and right axial view): Planning CT scan in arterial phase of the SRFA procedure with a 2,4 cm mass in liver segment VIIII and a 1, 7 cm mass on the superior pole of the right kidney. Maximum Intensity Projection (MIP) of the nonenhanced control CT scan showing the position of 9 coaxial needles and the position of 2 coaxial needles in the right kidney (right upper corner Fig. 3A).

The newly diagnosed lesions were interpreted as suspicious for metastatic disease (after CT, ultrasound, and MRI examination), and following an interdisciplinary team meeting, it was decided to perform the stereotactic radiofrequency ablation (SRFA) (Fig. 3) in December 2020.

SRFA is a minimal invasive therapeutic approach for the locally curative treatment of metastatic disease. In our institution, the intervention is performed under general anesthesia with muscle paralysis and patient immobilization on a CT table using either a single (Bluebag, Medica Intelligence Inc) or double vacuum fixation technique [[1], [2], [3], [4]]. Optical fiducials are applied to the skin of the thorax and upper abdomen for image registration, followed by a contrast-enhanced planning CT. To ensure precise and reproducible stereotactic conditions, the endotracheal tube (ETT) is temporarily disconnected during imaging, needle placement, and control imaging during the procedure. The pretreatment CT dataset is then transferred to an optically based navigation system (Stealth Station Treon Plus, Medtronic Inc) for planning SRFA probe trajectories. 15G coaxial needles are advanced through an aiming device, followed by control imaging. An unenhanced control CT is performed for verification of needle placement, and three 17G RF electrodes are introduced for serial tumor ablation. RF ablation is carried out using a Cool-tip RF generator with a standard ablation time or considering the increase in tissue impedance.

**Fig. 4:**
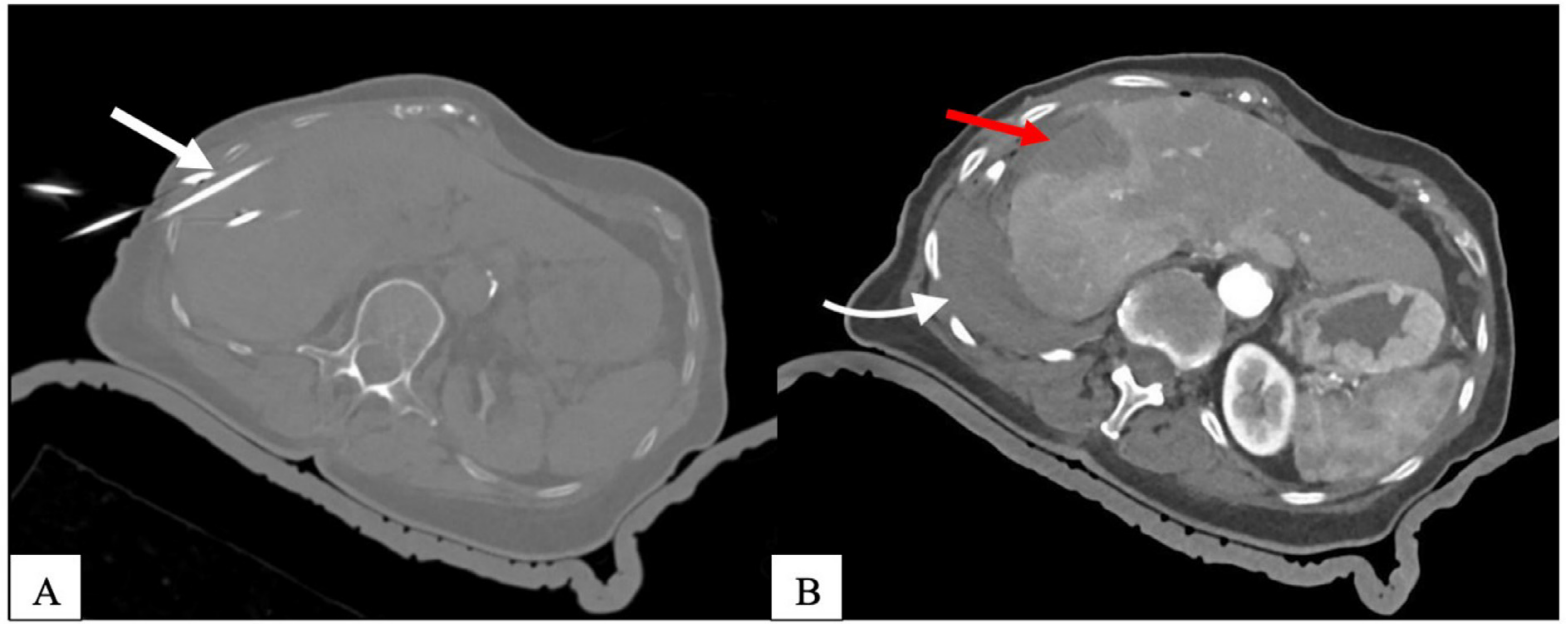
(A-B): CT scan during the coaxiales needles control and after ablation. (Figure 4A axial view) CT scan during the coaxiales needles control and after ablation (Figure 4B axial view) with corresponding necrosis zones in liver segment VIII. Notice the hemothorax (Figure 4B) on the right basal side of the Lung (white arrow).

Needle track ablation is performed to avoid bleeding and tumor seeding, and an immediate contrast-enhanced CT scan is conducted for verification of ablation zone size and assessment of possible complications. In this case, a postoperative CT scan showed successful ablation of the liver and kidney lesions (Fig. 4A).

However, an active extravasation of contrast media adjacent to the right basal pleura was detected, resulting in hematothorax of the ipsilateral side (Fig. 4B).

The patient suffered a hemorrhagic shock and pulseless electric activity for 5 minutes with spontaneous resuscitation.

**Fig. 5:**
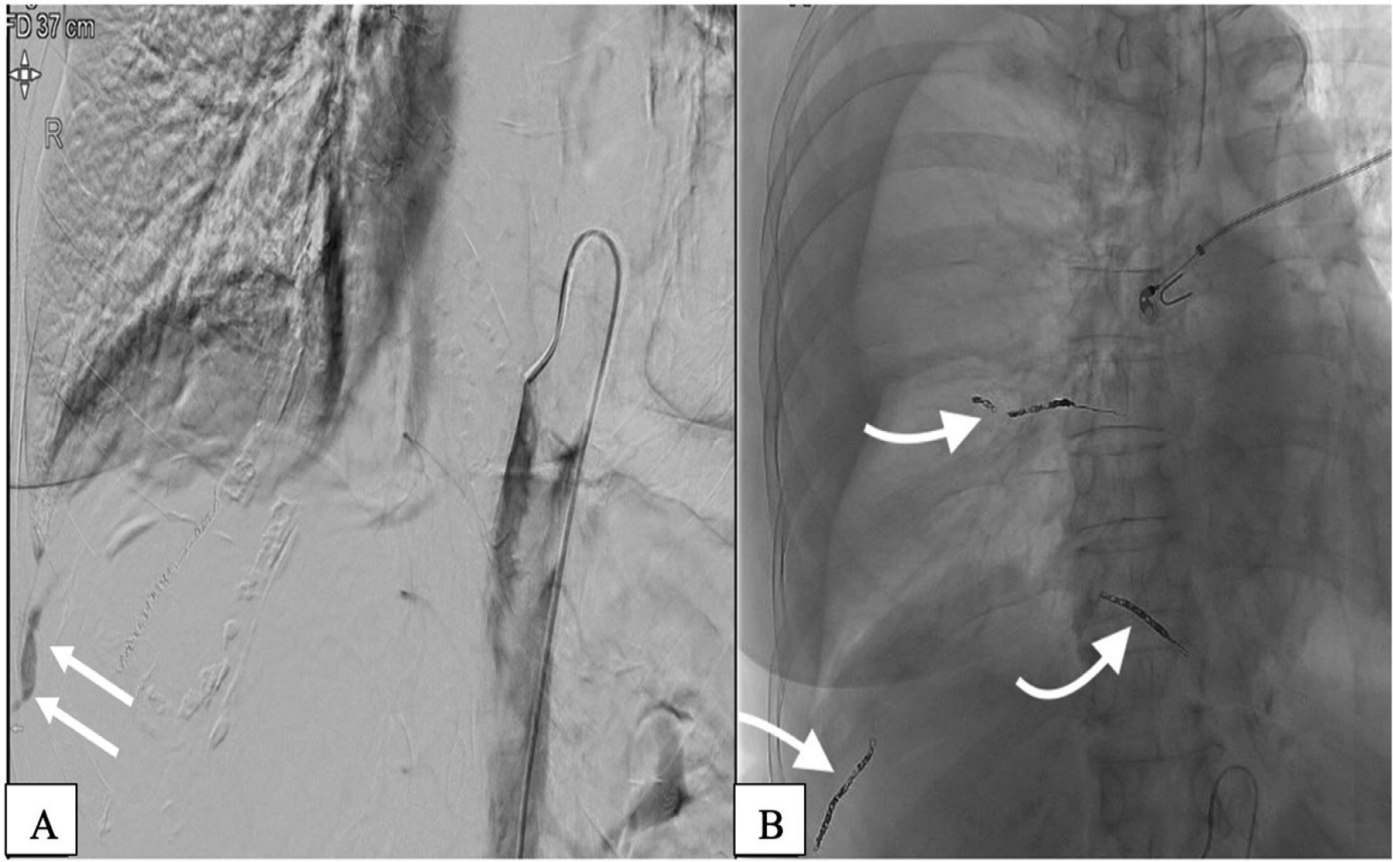
(A-B): Angiography of the anterior intercostal artery. 5A: Selective angiography of the anterior intercostal artery shows small focus of contrast extravasation the right intercostal arteries 9-11 (white arrows). 5B: Endovascular embolization of the intercostal artery 9-11 right (white arrows) using particles and microcoils. The angiogram shows no evidence of extravasation.

**Fig. 6:**
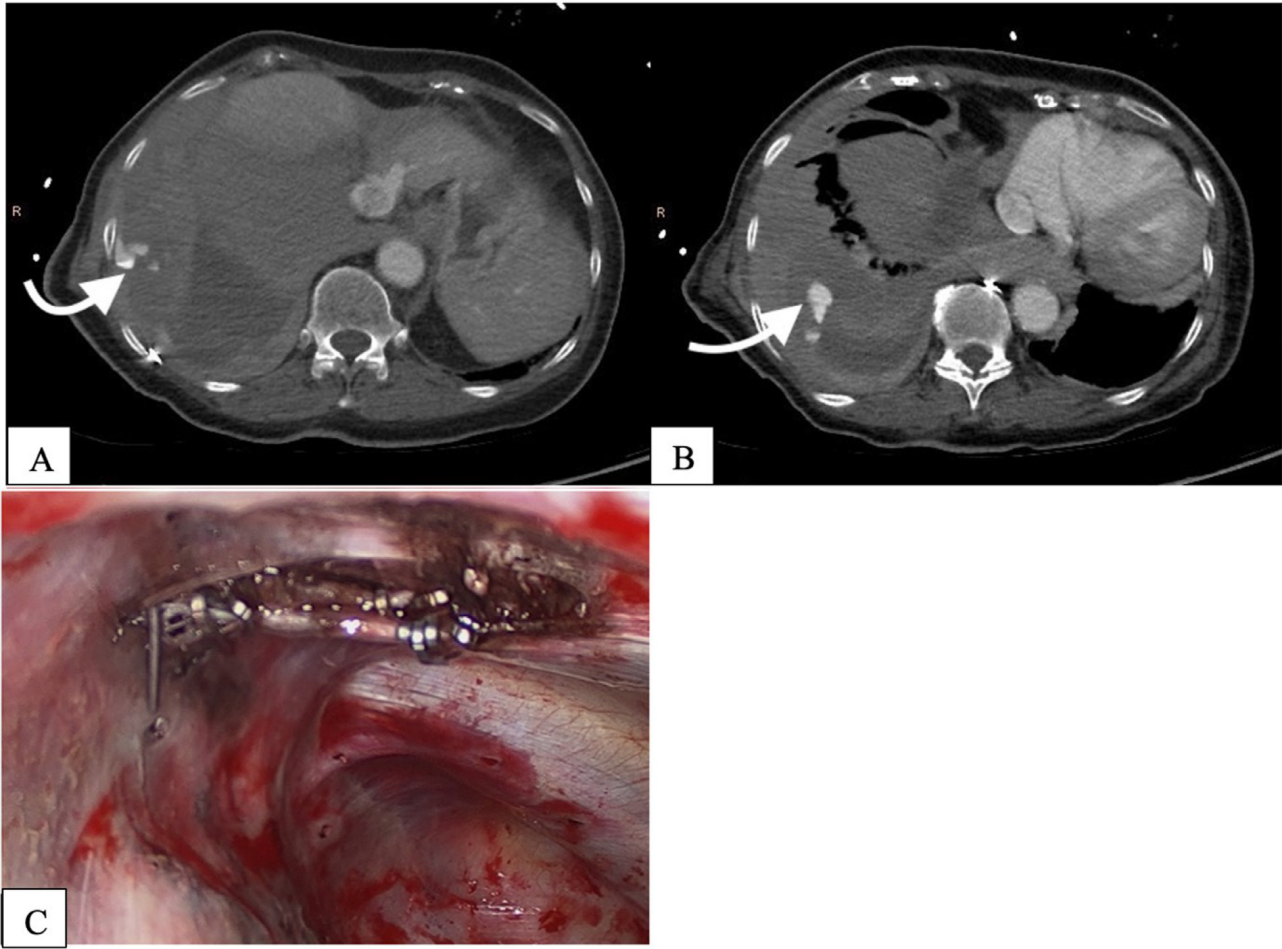
(A-C): A+B: Control CT-scan of the thorax shows persistent bleeding of 9th intercostal artery. C: Video-assisted thoracoscopy clipping of the intercostal artery.

The patient was immediately transferred to the angiography department, where active bleeding from the right intercostal arteries 9–11 (Fig. 5A) was diagnosed. Endovascular embolization of the right intercostal artery 9–11 was performed using particles and microcoils (Fig. 5B). In the angioscopy suite, the patient underwent immediate evacuation of the pleural hematoma with ultrasound-guided pigtail catheter insertion after the bleeding had been temporarily stopped by coil embolization at 4:30 PM However, on the same day (July 16, 2020), a further CT scan of the thorax (Figs. 6 A and B) was performed at 5:00 PM due to clinical signs of active bleeding. The CT scan revealed persistent bleeding from the 9th intercostal artery, requiring the insertion of a 28 CH Bülau drainage tube. Surgical evacuation of the hematoma and video-assisted thoracoscopic clipping of the intercostal artery were performed on the same day (Fig. 6C). After spending 2 weeks in the intensive care unit, the patient recovered and was discharged from the hospital in good condition.

**Fig. 7:**
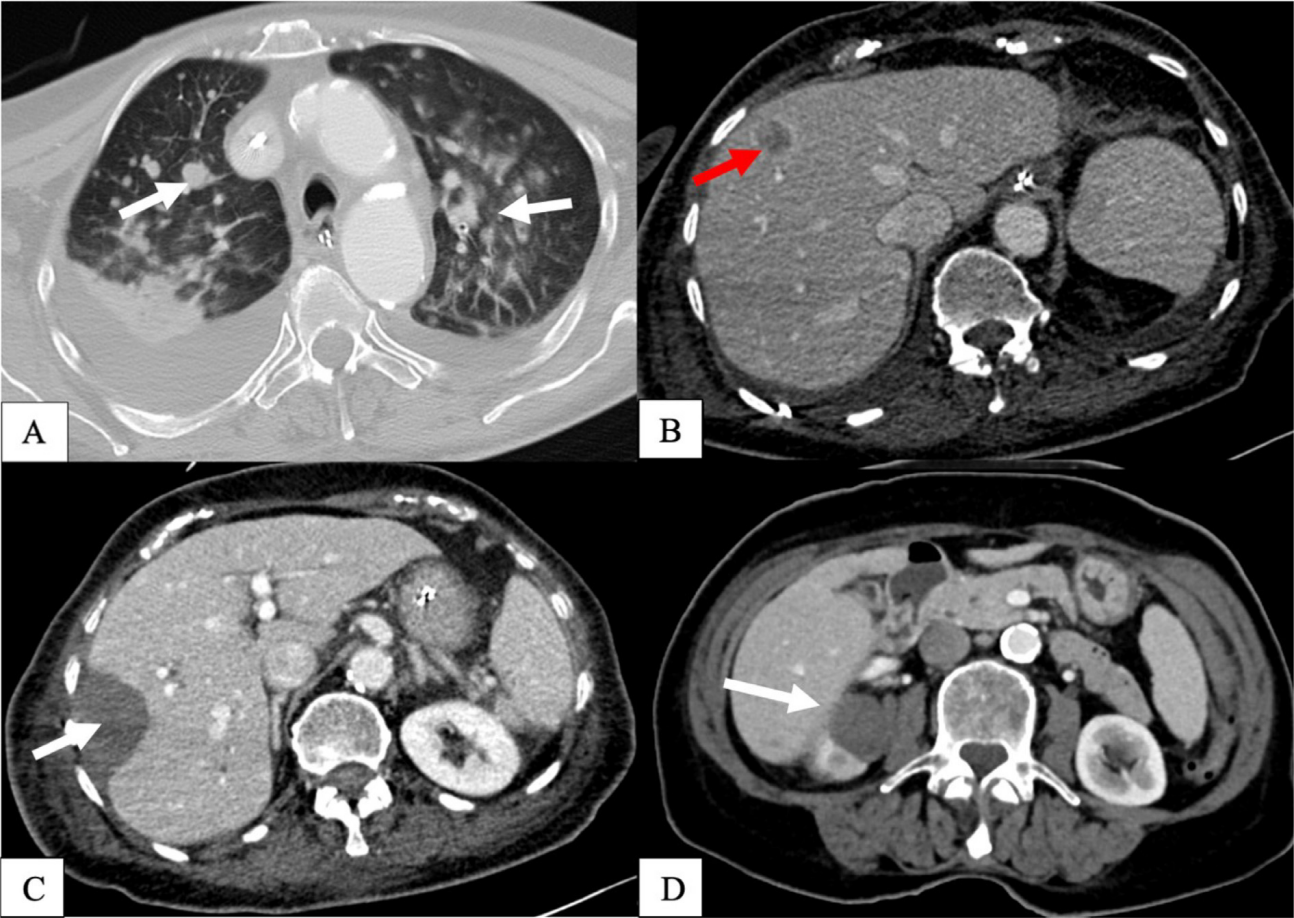
(A-D): Full-body CT scan 3 months after discharge from the hospital. 7A and 7B (axial view of Chest CT and Abdomen-CT) reveal hypodense lesions in the lungs and hypodense lesions with rim enhancement in liver segment VIII measuring 17 mm and in liver segment V measuring 14 mm, corresponding to new metastases. 7C and D (axial view of Abdomen and Kidney-CT) show no local recurrence of metastases adjacent to the ablation zone.

A follow-up full-body CT was performed on December 14, 2020, revealing multiple new pulmonary metastases and 3 new liver metastases (Figs. 7A and B). After SRFA, no local recurrence was detected adjacent to the ablation zone (Figs. 7C and D). The patient succumbed to spontaneous intracranial bleeding on December 16, 2020.

## Discussion

Nasal cavity MM is a rare form of melanoma that is highly aggressive and has a poor prognosis [[5], [6], [7], [8], [9]] and 3-year and 5-year survival of approximately 25%-18.2%, respectively [10]. MM is less common than cutaneous melanoma [11]. Any mucosal surface can be affected, although the majority usually arises from the head and neck's mucosa, anus and rectum, genital tract of the female, and urinary tract [12]. The primary sites of sinonasal mucosa melanoma`s involvement are the nasal septum, the nasal lateral wall, and the inferior nasal concha [13].

MM manifests most often in the 6th and 7th decades of life [14]. MM may be initially asymptomatic, but due to its expansive growth pattern, it may also cause obstruction and bleeding, depending on its location [[15], [16], [17]].

Usually, the symptoms develop late and are rather unspecific; therefore, MM is mostly diagnosed at an advanced stage [18].

The histopathologic appearance of MM may show a variable melanin deposit, epithelioid, or spindle-cell shape. The pathogenesis of mucosal melanoma is still not clear. Yet, no clear risk factors have been found for mucosal melanoma [19]. The most common genetic alterations in mucosal melanoma are B-raf protononcogene, serine/threonine kinase, and NRAS driver mutations. Specific immunohistochemical markers like S100, Melan-A, and HMB-45 are mostly used to confirm the diagnosis [20].

Melanomas appear as polypoid masses on CT with diffuse enhancement postcontrast [21]. MRI shows different signals based on melanin content. Lymph node metastasis is rare initially, but over half develop distant metastasis, commonly in the liver, lungs, bone, and brain. [[22], [23], [24]] 18F-FDG PET-CT scans aid in diagnosing residual tumor mass or distant metastasis [25]. The standard therapy for MM is a complete resection, which is challenging due to the aggressive recurrence of the disease. In metastatic disease, minimal invasive locoregional ablative treatment may be beneficial, depending on the extent and location of the disease. In our case, we performed minimally invasive locoregional SRFA of liver and kidney metastasis in one therapeutic session according to our standard setup, as previously described [2]. The advantages of a minimally invasive approach include the sparing of healthy tissue compared to open surgery and, generally, the reduction of adverse events. However, in this case, we effectively managed the complications resulting from SRFA of liver metastases, and the patient recovered within 2 weeks postoperatively.

As this is a report on a single case, long-term outcomes are not available, and further investigations are needed to evaluate the role of minimally invasive ablation techniques in the treatment of MM patients.

## Conclusion

Nasal cavity mucosal melanoma is a rare form of melanoma with a poor prognosis, primarily attributed to detection in an advanced stage. The standard therapy is complete resection, which is challenging due to the aggressive behavior and recurrence with early distant metastasis. In palliative cases, SRFA can pose a promising alternative therapeutic strategy, which is highlighted by our case presentation, with the primary goal of minimally invasive tumor reduction. The adverse events associated with the procedure were effectively managed by interventional radiology and surgery, and the patient recovered within 2 weeks from the procedure.

## Patient consent

Informed, written consent was obtained from the patient in accordance with COPE guidelines.

## References

[1] Bale R, Laimer G, Schullian P, Alzaga A (2023). Stereotactic ablation: a game changer?. J Med Imaging Radiat Oncol.

[2] Schullian P, Johnston EW, Putzer D, Laimer G, Waroschitz G, Braunwarth E (2021). Stereotactic radiofrequency ablation (SRFA) for recurrent colorectal liver metastases after hepatic resection. Eur J Surg Oncol.

[3] Bale R, Widmann G, Schullian P, Haidu M, Pall G, Klaus A (2012). Percutaneous stereotactic radiofrequency ablation of colorectal liver metastases. Eur Radiol.

[4] Putzer D, Schullian P, Bale R (2019). Locoregional ablative treatment of melanoma metastases. Int J Hyperthermia.

[5] Samstein RM, Carvajal RD, Postow MA, Callahan MK, Shoushtari AN, Patel SG (2016). Localized sinonasal mucosal melanoma: outcomes and associations with stage, radiotherapy, and positron emission tomography response. Head Neck.

[6] Konuthula N, Khan MN, Parasher A, Del Signore A, Genden EM, Govindaraj S (2017). The presentation and outcomes of mucosal melanoma in 695 patients. Int Forum Allergy Rhinol.

[7] Kepekci AH, Kig C, Gundogan GI (2018). The evaluation of malignant mucosal melanoma of nasal cavity with a rare occasion. Int J Physiol Pathophysiol Pharmacol.

[8] López F, Rodrigo JP, Cardesa A, Triantafyllou A, Devaney KO, Mendenhall WM (2016). Update on primary head and neck mucosal melanoma. Head Neck.

[9] Gras-Cabrerizo JR, Leon-Vintro X, Tarruella MM, Sarria GP, Gonzalez CB, Montserrat-Gili JR (2015). Management of sinonasal mucosal melanomas and comparison of classification staging systems. Am J Rhinol Allergy.

[10] Andrianakis A, Kiss P, Pomberger M, Wolf A, Thurnher D, Tomazic PV (2021). Sinonasal mucosal melanoma: treatment strategies and survival rates for a rare disease entity : A single center experience and review of literature. Wien Klin Wochenschr.

[11] Siegel R, Naishadham D, Jemal A (2012). Cancer statistics, 2012. CA Cancer J Clin.

[12] Mihajlovic M, Vlajkovic S, Jovanovic P, Stefanovic V (2012). Primary mucosal melanomas: a comprehensive review. Int J Clin Exp Pathol.

[13] Clifton N, Harrison L, Bradley PJ (2011). Malignant melanoma of nasal cavity and paranasal sinuses: report of 24 patients and literature review. J Laryngol Otol.

[14] McLaughlin CC, Wu XC, Jemal A, Martin HJ, Roche LM, Chen VW (2005). Incidence of noncutaneous melanomas in the U.S. Cancer..

[15] Dauer EH, Lewis JE, Rohlinger AL, Weaver AL, Olsen KD (2008). Sinonasal melanoma: a clinicopathologic review of 61 cases. Otolaryngol. Head Neck Surg..

[16] Roth TN, Gengler C, Huber GF, Holzmann D (2010). Outcome of sinonasal melanoma: clinical experience and review of the literature. Head Neck.

[17] Vandenhende C, Leroy X, Chevalier D, Mortuaire G (2012). Sinonasal mucosal melanoma: retrospective survival study of 25 patients. J Laryngol Otol.

[18] Patrick RJ, Fenske NA, Messina JL (2007). Primary mucosal melanoma. Journal of the American Academy of Dermatology.

[19] Jasper P, Jungbauer WN, Poupore NS, Nguyen SA, Howell J, Neville BW (2022). Mucosal melanoma in situ of the oral cavity: a case report and systematic review of the literature. Turk Arch Otorhinolaryngol.

[20] X Green B, Elhamshary A, Gomez R, Rahimi S, Brennan PA (2017). An update on the current management of head and neck mucosal melanoma. J Oral Pathol Med.

[21] Eggesbø HB (2012). Imaging of sinonasal tumours. Cancer Imaging.

[22] Nardi C, Vignoli C, Vannucchi M, Pietragalla M (2019). Magnetic resonance features of sinonasal melanotic mucosal melanoma. BMJ Case Rep.

[23] Andrianakis A, Kiss P, Pomberger M (2021). Sinonasal mucosal melanoma: treatment strategies and survival rates for a rare disease entity. Wien Klin Wochenschr.

[24] Clifton N, Harrison L, Bradley PJ (2011). Malignant melanoma of nasal cavity and paranasal sinuses: report of 24 patients and literature review. J Laryngol Otol.

[25] Haerle SK, Soyka MB, Fischer DR (2012). The value of 18F-FDG-PET/CT imaging for sinonasal malignant melanoma. Eur Arch Otorhinolaryngol.

